# Radiation Damage Mechanisms of Chemotherapeutically Active Nitroimidazole Derived Compounds

**DOI:** 10.3389/fchem.2019.00329

**Published:** 2019-05-14

**Authors:** Jacopo Chiarinelli, Anna Rita Casavola, Mattea Carmen Castrovilli, Paola Bolognesi, Antonella Cartoni, Feng Wang, R. Richter, Daniele Catone, Sanja Tosic, Bratislav P. Marinkovic, Lorenzo Avaldi

**Affiliations:** ^1^CNR-Istituto di Struttura Della Materia (CNR-ISM), Area della Ricerca di Roma 1, Monterotondo Scalo, Italy; ^2^Dipartimento di Scienze, Università di Roma Tre, Rome, Italy; ^3^Dipartimento di Chimica, Sapienza Università di Roma, Rome, Italy; ^4^Molecular Modelling Discovery Laboratory, Department of Chemistry and Biotechnology, Faculty of Science, Engineering and Technology, Swinburne University of Technology, Melbourne, VIC, Australia; ^5^Elettra-Sincrotrone Trieste, Trieste, Italy; ^6^CNR-Istituto di Struttura Della Materia, Area della Ricerca di Tor Vergata, Rome, Italy; ^7^Institute of Physics, Laboratory for Atomic Collision Processes, University of Belgrade, Belgrade, Serbia

**Keywords:** nitroimidazole, radiosensitizers, mass spectrometry, PEPICO experiments, appearance energy, DFT

## Abstract

Photoionization mass spectrometry, photoelectron-photoion coincidence spectroscopic technique, and computational methods have been combined to investigate the fragmentation of two nitroimidazole derived compounds: the metronidazole and misonidazole. These molecules are used in radiotherapy thanks to their capability to sensitize hypoxic tumor cells to radiation by “mimicking” the effects of the presence of oxygen as a damaging agent. Previous investigations of the fragmentation patterns of the nitroimidazole isomers (Bolognesi et al., 2016; Cartoni et al., 2018) have shown their capacity to produce reactive molecular species such as nitric oxide, carbon monoxide or hydrogen cyanide, and their potential impact on the biological system. The results of the present work suggest that different mechanisms are active for the more complex metronidazole and misonidazole molecules. The release of nitric oxide is hampered by the efficient formation of nitrous acid or nitrogen dioxide. Although both metronidazole and misonidazole contain imidazole ring in the backbone, the side branches of these molecules lead to very different bonding mechanisms and properties.

## Introduction

The use of “high-throughput screening” methods for drug discovery allows to rapidly conduct a very broad and random screening over an enormous number of chemicals. However, in these procedures the very fundamental chemical and physical mechanisms that determine the activity of these compounds at the molecular level remain unknown. On the other hand, highly sensitive experimental techniques and accurate computational methods have been developed to provide a detailed description of model molecules and to link their electronic and geometric structure to their functions. This, in particular, is of paramount importance in the case of the molecular response of cells and their building blocks to radiosensitising drugs used to increase the potential of radiotherapy. Despite the fact that the typical energies used in radiotherapy are in the keV to MeV range it is well-documented (García Gómez-Tejedor and Fuss, 2012) that a large fraction of the radiation damage on biological systems is due to secondary processes releasing particles (electrons, ions, radicals) with a broad energy distribution, which can subsequently trigger the damaging of DNA and its surrounding environment. Slow electrons with energy of a few eV have been considered among the most active species (Boudaiffa et al., 2000; Michael and O'Neill, 2000). Thus, the potential impact of VUV based techniques is due to their possibility to provide detailed information on the electronic structure and fragmentation of valence orbitals, which are the ones mainly involved in the processes induced by low energy electrons. Photoelectron-photoion coincidence, PEPICO, experiments then, due to their energy selectivity, provide detailed insights on state-selected fragmentation, and therefore are particularly suited to identify the states involved in the production of specific fragments and the release of radicals.

The question is whether the fragmentation mechanisms and properties identified in the model systems are still active at macroscopic level in more complex and realistic systems.

**Figure 1:**
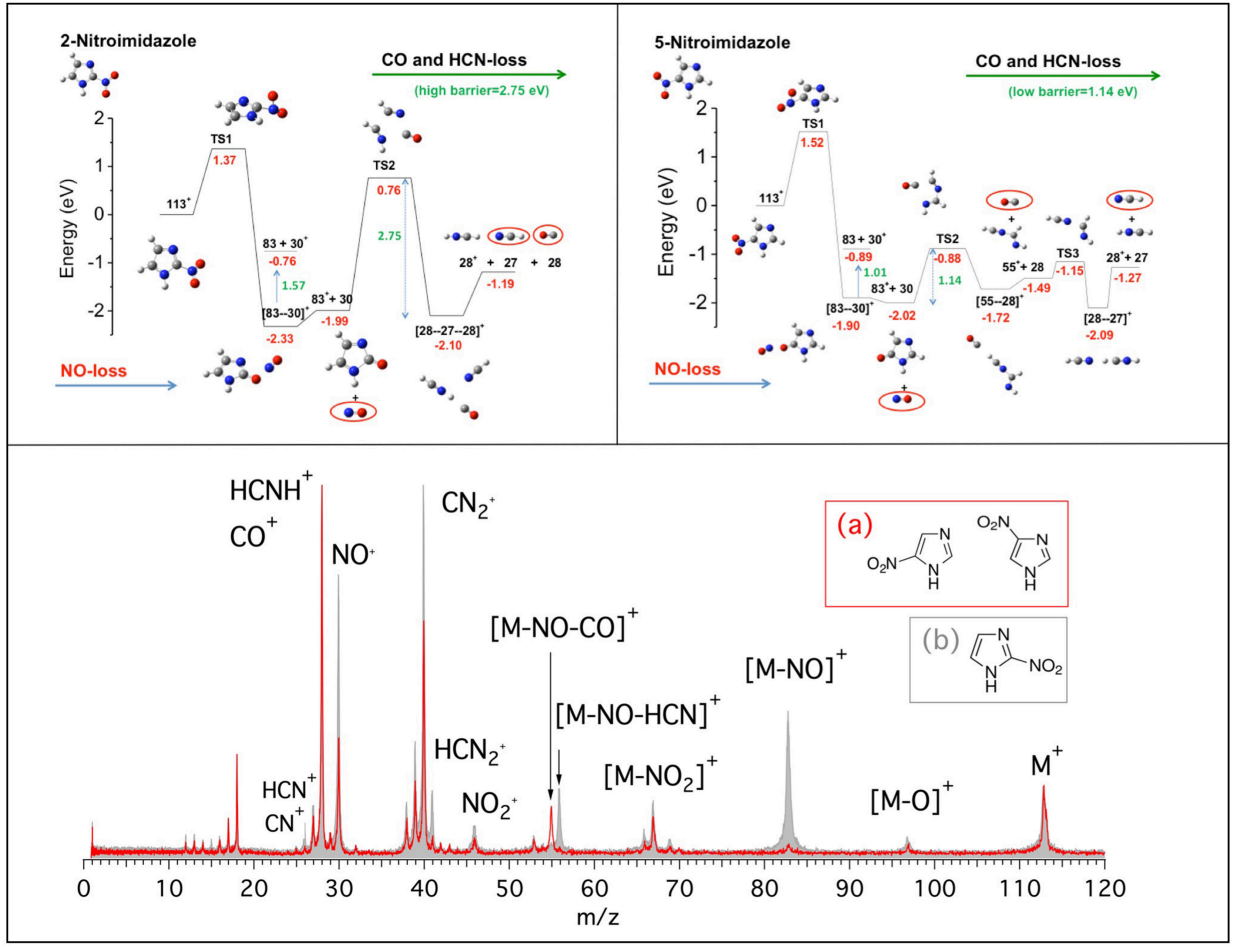
(Bottom panel) Mass spectra of 4(5)-NI [red line and inset **(A)**] and 2-NI molecules [gray, full area, and inset **(B)**] measured at 60 eV photon energy. The assignment of the main fragments is reported. (Top panels) Potential energy surfaces of the 2-NI and 5-NI, respectively, for the fragmentation of their corresponding molecular ions M^+^ (m/z 113) calculated at the CCSD/6-311++G**//B3LYP/6-311++G** level of theory (Bolognesi et al., 2016). The molecular ion M^+^ as well as the fragments [M-O]^+^, ${\text{NO}}_{2}^{+}$, ${\text{HCN}}_{2}^{+}$, NO^+^, and HCNH^+^ are all radical ions; the radical symbol • as been omitted here and all over the paper for sake of simplicity.

In this work we present the results of a bottom-up approach, which goes from the model molecule to the real drugs used in therapy. Photoionization mass spectrometry (PIMS), photoelectron spectroscopy and photoelectron-photoion spectroscopic (PEPICO) technique, and computational methods have been combined to investigate nitroimidazole (NI) derived molecules. These molecules are used in radiotherapy thanks to their capability to sensitize hypoxic tumor cells to radiation by “mimicking” the effects of the presence of oxygen as a damaging agent (Wardman et al., 2007; Rockwell et al., 2009; Sonveaux et al., 2009; Wilson and Hay, 2011; Higgins et al., 2015). However, the detailed mechanisms of their operation at molecular levels are still unknown, making difficult any rationale in the design of more efficient and less toxic drugs for treatment. In our bottom-up approach we have investigated the building blocks of the molecules used in therapy: the 2- and 4(5)-NI molecules (Bolognesi et al., 2016; Cartoni et al., 2018). The main results of the investigation of the fragmentation patterns of the NI isomers are summarized in Figure 1 where the mass spectra (bottom panel) of the 2-NI and 4(5)-NI and the potential energy surfaces (top panels) for the NO loss and further fragmentations are shown.

At first glance, the most evident observation in Figure 1 is that the fragment at m/z 83 is one of the leading fragmentation channels in 2-NI, while it is almost absent in the 4(5)-NI sample. The m/z 83 fragment can be unambiguously attributed to the loss of nitric oxide, which is particularly relevant for its potential implications in the biological context due to the well-recognized action of NO as a radiosensitiser and vasodilator (Wardman et al., 2007; Rockwell et al., 2009; Sonveaux et al., 2009). This may suggest that 2-NI is able to release a significantly larger amount of NO than 4(5)-NI, hence supporting the observation of its higher efficiency as radiosensityzer (Wardman et al., 2007). The quantum mechanical calculations (Figure 1, top panels) at the B3LYP/6-311++G^**^ level of theory for the geometry optimization and at the CCSD/6-311++G^**^ level for single-point energy calculation show that all of the nitroimidazole isomers are likely to release NO. However, in 4(5)-NI the subsequent fragmentation of the residual m/z 83 intermediate breaks the imidazole ring releasing HCN and CO molecules, while in 2-NI the higher kinetic stability of the ring leaves the intermediate intact. These results explain the different intensity of the fragments at m/z 83, 55, 30, and 28 observed in the mass spectra of both 2-NI and 4(5)-NI, respectively. From these evidences we determined that all the nitroimidazole isomers release the NO fragment with similar mechanisms. The released NO, being active for a short period of time after irradiation, could act by fixing dangling bonds in damaged DNA, making the damage permanent and by “favoring either drug delivery or the therapeutic efficacy of irradiation through transient tumor reoxygenation,” as suggested by Sonveaux et al. (2009). In addition to the redox mechanism, this could provide explanation for the potential of all nitroimidazoles as radiosensitisers active on hypoxic tumors (Higgins et al., 2015). On the other hand, the release of carbon monoxide, CO, and hydrogen cyanide, HCN, more pronounced in 4(5)-NI isomers, may induce an opposite effect by efficiently attaching to hemoglobin (Berg et al., 2012) and inhibiting the *cytochrome c* oxidase in mitochondria (Yoshikawa and Caughey, 1990), respectively. Therefore, this effectively reduces the needed oxygenation and the overall radiosensitising effect.

**Scheme 1:**
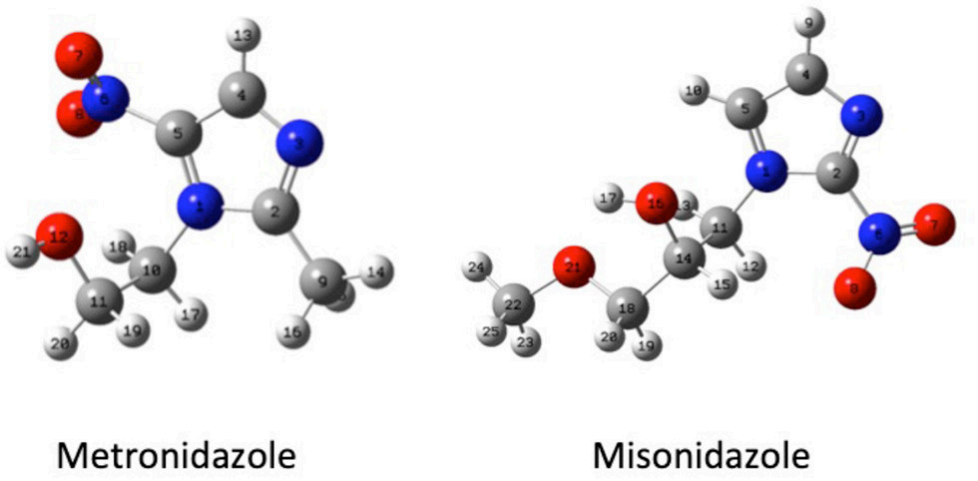
The geometry (structure) optimized at B3LYP/6-311++G** level of the metronidazole (left panel) and misonidazole (right panel) molecules. The labeling of each atom in the molecules is also shown.

Guided by these former results, in this work we have studied both experimentally and theoretically the fragmentation mechanisms of metronidazole [IUPAC name: 2-(2-methyl-5-nitro-1H-imidazol-1-yl) ethanol] and misonidazole [IUPAC name (RS)-1-methoxy-3-(2-nitroimidazol-1-yl)propan-2-ol] (Scheme 1), the two radiosensitisers built on the 5-NI and 2-NI compounds, respectively, which are used in radiotherapy.

The experimental methods are described in section Experimental, while the theoretical ones are summarized in section Theoretical Methods. The experimental results are analyzed in section Results and discussed/interpreted with the support of the DFT theoretical calculations in section Discussion. Finally some conclusions are presented in section Conclusion. In the following the radical symbol • has been omitted for the sake of simplicity.

## Experimental

The experiments have been performed at the Circular Polarized (CIPO) and Gas Phase Photoemission (GAPH) beamlines of the Elettra synchrotron radiation source, Trieste (Italy). The characteristics of the beamlines have been described in details elsewhere (Derossi et al., 1995; Blyth et al., 1999) and will not be repeated here.

The metronidazole sample of analytical standard was purchased from Sigma-Aldrich, while the misonidazole one with 95% purity by Vinci-Biochem Srl. Both samples have been used without further purification. They are in the form of powders at standard ambient temperature and pressure, and they are evaporated in a furnace at about 150°C. No evidence of sample dissociation is observed.

The Appearance Energy, AE, of the different fragments has been obtained by the measurement of the photoionization efficiency curves of the parent ion and selected fragments at the CIPO beamline using the aluminum normal incidence monochromator (NIM), that covers the photon energy range 5–17 eV with a resolving power of about 1,000. The setup consists of five electrostatic lenses that focus and accelerate the ions from the region of interaction to the quadrupole mass spectrometer (QMS). This is a commercial QMS (10–4000 u, Extrel 150-QC 0.88 MHz) with a mass resolution M/ΔM of about 500. It is mounted perpendicularly to the photon beam and to the gas source. The photoionization efficiency curves were normalized to the photon intensity, measured simultaneously by a photodiode located at the end of the beamline. The photon energy was calibrated against the autoionization features observed in the Ar total photoionization spectrum between the 3p spin orbit components. In the photon energy scans up to 11.7 eV, a lithium fluoride filter was used to remove the second order radiation. Above this energy, the contribution of the second order radiation was evaluated by comparing the Ar^+^ ion yield measured as a function of the photon energy to its ionization cross section (Marr and West, 1976). This second order contribution has been taken into account in the extraction of the photoionization efficiency curves (Castrovilli et al., 2014).

**Figure 2:**
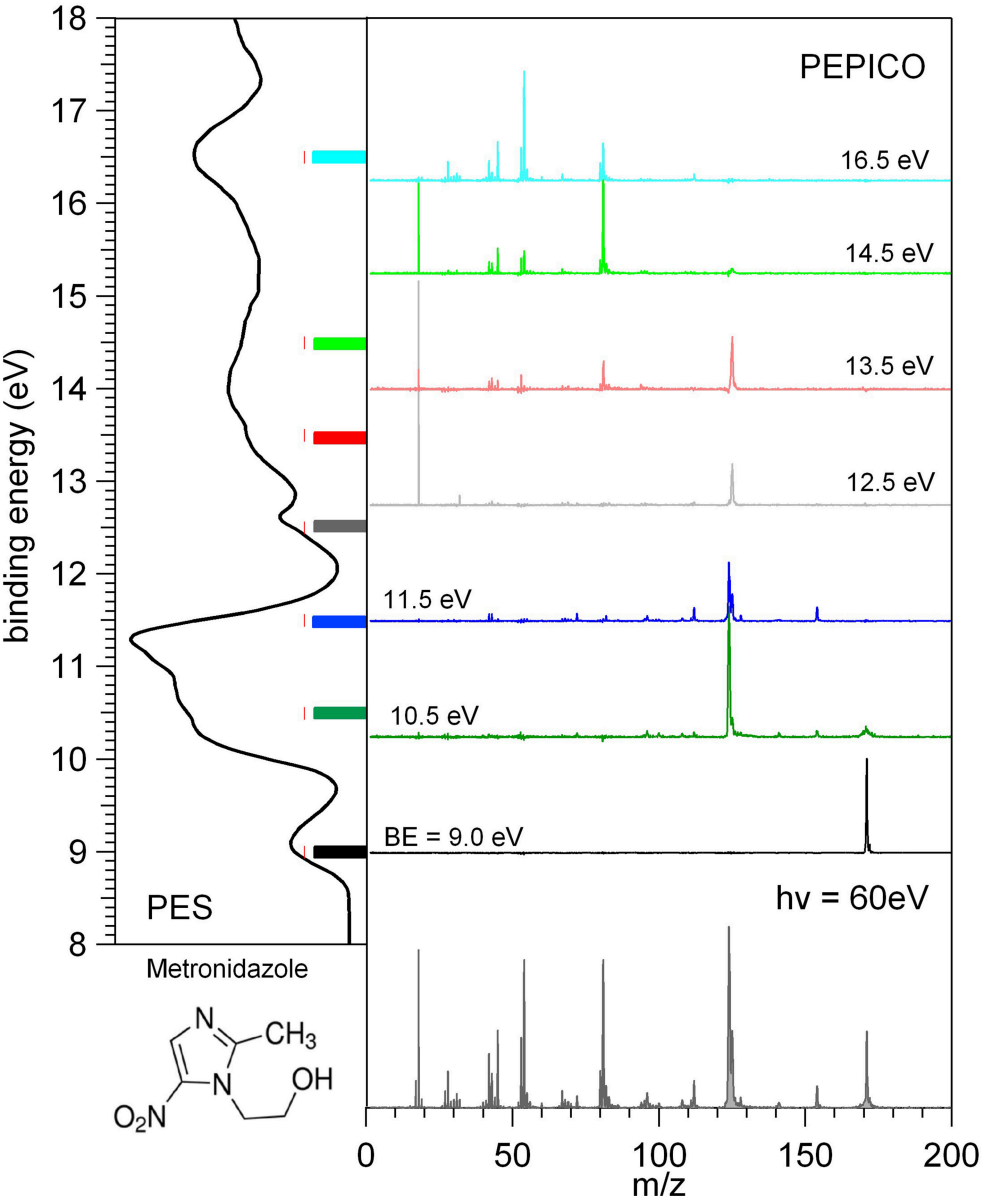
Photoelectron spectrum (left panel), photoionization mass spectrum (bottom panel), and a set of PEPICO spectra (central panel) of metronidazole. The color code of the PEPICO spectra corresponds to the colored bars in the PES spectrum, which identify the different regions of the spectrum selected by the detection of the energy selected photoelectrons.

The photoelectron and mass spectra as well as the photoelectron-photoion coincidence, PEPICO, spectra have been measured at the GAPH beamline using a high vacuum chamber hosting a hemispherical analyzer (VG 220i) equipped with six channeltron dectectors and a custom made Wiley McLaren (Wiley and McLaren, 1955) time-of-flight (TOF) mass spectrometer mounted opposite to each other at the magic angle with respect to the polarization axis of the photon beam. The TOF mass spectrometer, working in conjunction with the “virtually” continuous ionization source provided by the multibunch operation mode of the synchrotron radiation, is operated in pulsed extraction mode. The repeller and extractor electrodes are polarized with antisymmetric voltages (manufacturer Directed Energy Inc., model PVM4210) driven by an external trigger, which provides a typical extraction field of 700 V/cm. The electron and ion mass analyzers can be operated independently, for photoelectron spectroscopy and photoionization mass spectrometry, respectively. In these operation modes (i) the hemispherical analyzer is normally operated with pass energy of 5 eV, corresponding to a kinetic energy resolution of about 150 meV or (ii) the extraction field of the TOF spectrometer is triggered using a 1 kHz pulse generator (Stanford Research Systems DG535) to extract the ions. The two analyzers can also be operated simultaneously for coincidence measurements. In this mode a residual penetration field from the drift tube of the TOF produces a kinetic energy shift of the photoelectron spectrum (easily taken into account by the calibration procedure) and a degradation of the energy resolution of the electron analyzer. Therefore, in the coincidence mode the electron energy analyzer has been operated at pass energy of 20 eV, with a gain in efficiency, but no further loss of resolution. The final energy resolution is estimated to be around 0.5 eV. In order to perform photoelectron-photoion coincidence spectroscopic measurements, the electronic chain schematically reported in Figure 1 of Plekan et al. (2008) has been used for all detectors of the VG analyser. The three different types of measurement, that can be performed by this set-up, are illustrated in Figure 2 where the photoelectron spectrum, the photoionization mass spectrum and a few PEPICO spectra of metronidazole measured at 60 eV photon energy are shown.

In the PEPICO measurements the selection of the kinetic energy of the detected photoelectron allows to make a state selected investigation of the fragmentation of the molecule as clearly shown in the central panel of Figure 2. From the spectra measured at different binding energies, BE, the branching ratio for the formation of a selected fragment vs. photon energy can be obtained. The procedures for the treatment of the PEPICO spectra, with the subtraction of the background due to the random coincidence and relative normalization have been described recently elsewhere (Chiarinelli et al., 2018) and therefore will not be repeated here.

In Figure 2 each energy selected mass spectrum is characterized by only a few fragments as compared to the unselected mass spectrum (see bottom panel in Figure 2); the parent ion is observed only near the ionization energy of the molecule; only a few fragmentation channels at a time are associated with a selected electronic state of the cation although lower energy fragmentation channels are already energetically open.

## Theoretical Methods

Quantum chemical calculations have been performed with Density Functional Theory (DFT). The geometries were optimized using the Becke, three-parameter, Lee-Yang-Parr (B3LYP) functional with the 6-311++G^**^ basis set. The frequency analysis was based on the normal mode harmonic approximation (Wong, 1996). All critical points were characterized as energy minima or transition state structures (TS) by calculating the harmonic vibrational frequencies at the same level of theory. They were also used to compute the zero-point and thermal energy corrections. The TS were unambiguously related to their interconnected energy minima by intrinsic reaction coordinates (IRC) calculations (Gonzalez and Schlegel, 1989, 1990).

The outer valence vertical ionization energies were calculated using the outer valence Green function OVGF/6-311++G^**^ methods (von Niessen et al., 1984; Ortiz, 1988), based on the optimized geometries using B3LYP/6-311++G^**^.

**Figure 3:**
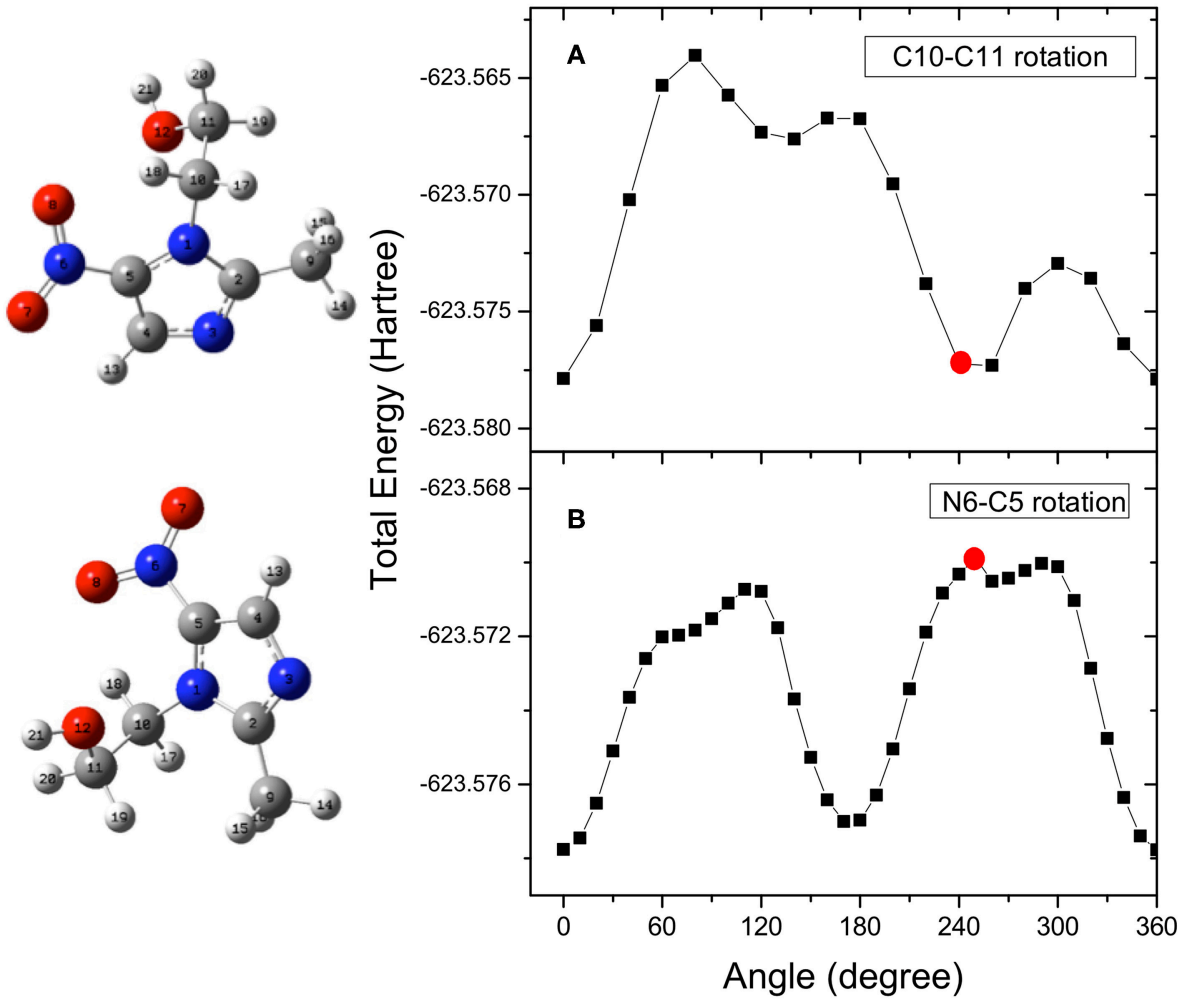
Potential energy surface for rotation of the metronidazole molecule around the N6-C5 (dihedral angle O7N6C5C4) **(A)** and C10-C11 (dihedral angle O12C11C10N1) **(B)**, respectively. The red dots indicate the angle of rotation of the structure represented on the left hand side of the figure. They have been chosen as examples of the effect of the rotation on the structure.

The determination of the potential energy surfaces for the two flexible metronidazole and misonidazole compounds is very challenging, because rotations of their single C-N, C-C, and C-O bonds may produce a number of local minimum structures, i.e., conformers on their potential energy surfaces. All the possible stable conformers of a molecule may contribute to the measurement, based on Boltzmann's distribution at the temperature of the measurement. The search for all possibly stable conformers can be a daunting task. The structures and vibrational frequencies of the different structures in the potential energy surface of metronidazole and misonidazole for the electronic ground state were calculated using the Gaussian 09 program package (Frisch et al., 2009). High-level ab initio calculations have been done to map the potential energy surface for internal rotation of the molecules. Among the several rotational paths that the molecule can follow, here we have considered the ones which, leading to large potential energy barriers, are able to produce stable local minimum structures, i.e., conformers of the compounds. These rotational pathways are represented in the following figures. In the case of metronidazole the rotations around the N6-C5, which is the nitro and imidazole N-C bond and C10-C11 which is the ethanol backbone C-C bond, exhibit apparent energy barriers due to formation of the intramolecular hydrogen bonding. The relative potential energy scans were calculated rotating these two bonds, N6-C5 (dihedral angle O7N6C5C4), and C10-C11 (dihedral angle O12C11C10N1), respectively. The potential energy scans as a function of the dihedral angles are reported in Figure 3.

Optimizations of the structures are performed again at the local minima structures on the potential energy surface and the stable local minimum structures (conformers) are then found. The selected parameters which characterize the minimum structures conformer I (C10-C11) and conformer II (N6-C5) are collected in Table 1SM. We found that although conformers I and II obtained from rotation of the N6-C5 and C10-C11 bonds, respectively, are almost energy degenerate with a difference of 0.085 KJ/mol, they are different conformers as other properties such as dipole moments are very different. Vibrational frequency calculations were performed at the same level of the geometry optimization to characterize the stationary points as either minima or transition state structures (first-order saddle points). The frequencies calculated for the minimum structure conformers are all positive confirming that they are true minimum structures.

**Table 1:** Experimental and theoretical values calculated at the B3LYP6-311++G^**^ level of the ionization potential, IP, of the parent ion and AE of some fragments of the metronidazole, and misonidazole.

**Metronidazole**				**Misonidazole**				
**m/z**	**Assignment fragments**	**Exp**.	Exp^*^	**Theory**	**m/z**	**Assignment**	**Exp**.	**Theory**
171	M = C_6_H_9_N_3_O_3_	8.57 ± 0.05	8.84 ± 0.1	8.6	201	M = C_7_H_11_N_3_O_4_	8.55 ± 0.03	8.77
125	M-NO_2_	9.33 ± 0.07	10.02 ± 0.1	9.59	155	M-NO_2_	8.90 ± 0.04	9.1
124	M-HONO	9.14 ± 0.10	9.96 ± 0.1		154	M-HONO	8.91 ± 0.04	
81	M-NO_2_-(T_1_-H)	12.22 ± 0.10						
54	M-NO_2_-(T_1_-H)-HCN	13.42 ± 0.10		13.55				
					45	CH_2_OCH_3_	10.21 ± 0.10	10.40
45	T_1_ = CH_2_CH_2_OH	12.09 ± 0.10		11.34				

*In the case of metronidazole also the experimental data by Guo et al. (2012) have been reported*.

* (Guo et al., 2012)

**Figure 4:**
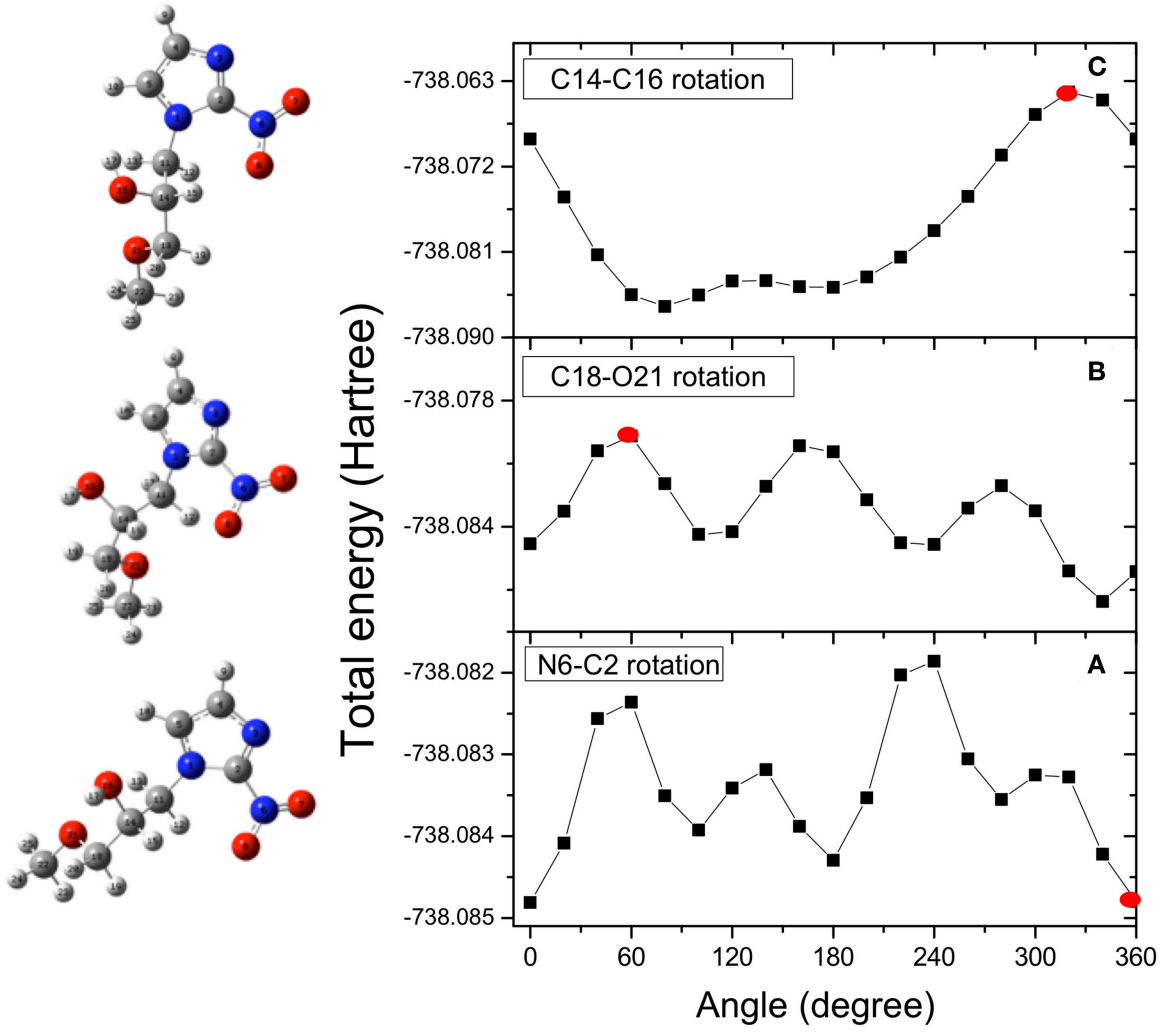
Potential energy surfaces for rotation of the misonidazole molecule around the N6-C2 (dihedral angle O7N6C2N3) **(A)**, C18-O21 (dihedral angle C14C18O21C22) **(B)** and C14-O16 (dihedral angle H17O16C14C11) **(C)** bonds, respectively. The red dots indicate the angle of rotation of the structure represented on the left hand side of the figure. They have been chosen as examples of the effect of the rotation on the structure.

In the case of misonidazole the same high-level ab-initio calculations have been performed to map out the potential energy surface for internal rotation of the NO_2_ group around the N6-C2 bond (Figure 4A), of the OCH_3_ group around the C18-O21 bond (Figure 4B) and of the OH group around the C14-O16 bond(Figure 4C).

Once the minimum in the potential energy surface is found, we optimized it for the most stable structure. Due to intramolecular hydrogen bonding, the rotation of the OH group produces a conformer more stable than the rotation around the other bonds. As in the case of metronidazole, the energy differences between the analyzed conformers are in the range of few Kcal/mol. It means that already at room temperature there is a mix of different conformers. In the calculations only one structure, the most stable one resulting from the rotation of the C14-C16 bond (dihedral angle H17O16C14C11), is chosen as starting point of the fragmentation pathway. This may represent a strong limit, because some pathways might not be identified due to a different initial structure.

## Results

**Figure 5:**
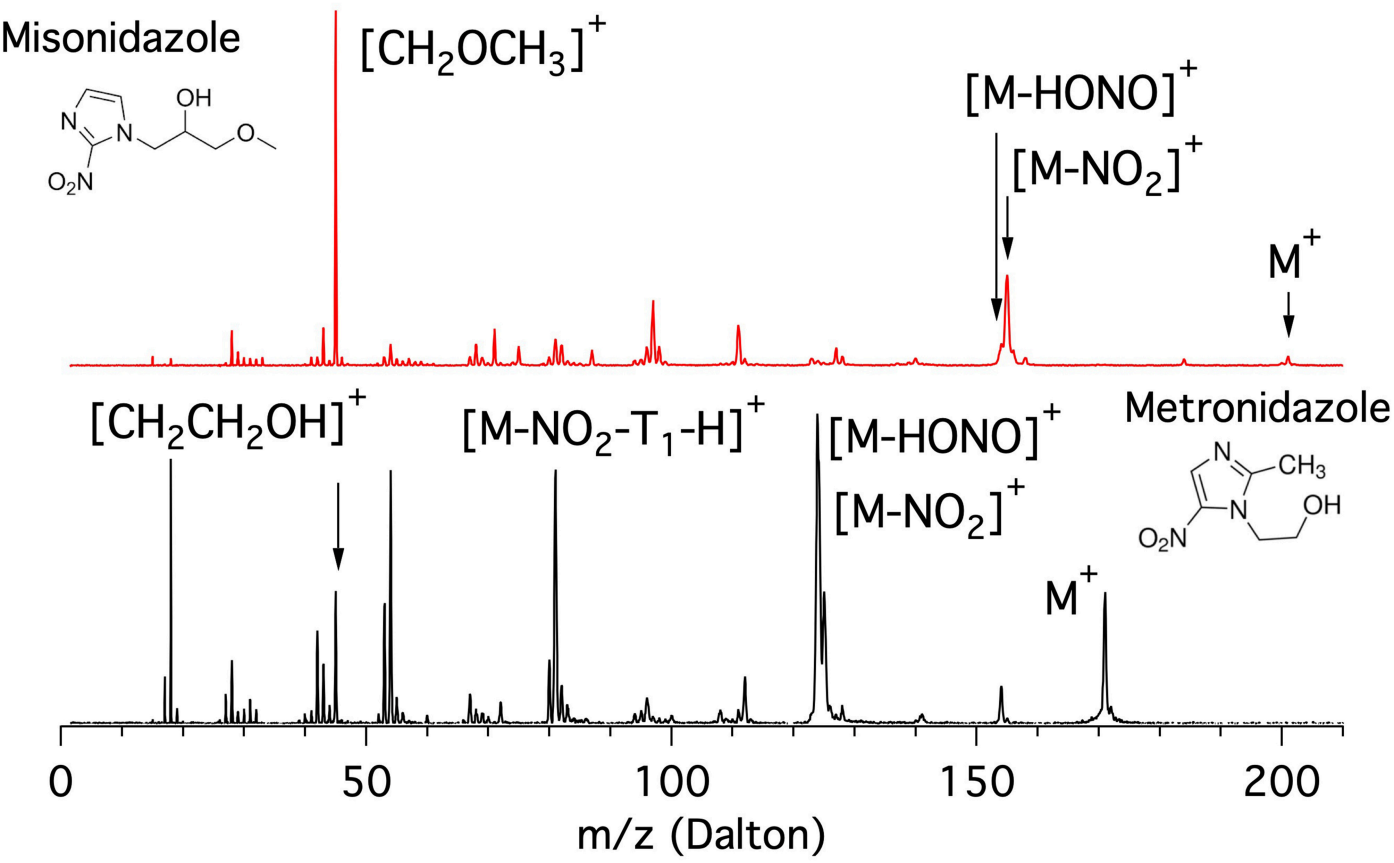
Mass spectra of the misonidazole (top panel) and metronidazole (bottom panel) molecules measured at 60 eV photon energy.

The mass spectra of the metronidazole and misonidazole molecules measured at 60 eV photon energy are shown in Figure 5.

The metronidazole molecule is built on the 5-NI where the H bound to C2 is replaced by the methyl group CH_3_ and the one bound to the N1 in the imidazole ring by a “short” ethanol tail (T_1_ = CH_2_CH_2_OH, m = 45 Da) terminated by a OH group. In its mass spectrum (bottom panel of Figure 5) the main features are at m/z 171 (parent ion M^+^), m/z 125, and 124 ([M-NO_2_]^+^ and [M-HONO]^+^, respectively), around m/z 81 ([M-NO_2_-(T_1_-H)]^+^ group), m/z 53 (corresponding to the opening of the imidazole ring following a further fragmentation of m/z 81), and around m/z 42 with the peak at m/z 45 assigned to the ethanol cation tail [T_1_]^+^. Below m/z 20 the peaks due to smaller fragments such as H_2_O and CH_3_ are also observed.

The misonidazole molecule is built on the 2-NI where the H bound to the N1 of the imidazole ring is replaced by a “longer” tail (T_2_ = CH_2_CH(OH)CH_2_OCH_3_, m = 89 Da) in which the propanol-2 is terminated by a methoxy group. The inspection of the mass spectra of misonidazole (top, red) and metronidazole (bottom, black) given in Figure 5 shows that, although the two molecules share some common fragment groups, the relative intensities of the different fragments are very different. Two major differences are observed. One major difference in the two spectra is represented by the near absence of the parent ion in misonidazole; the other is that the very limited number of relevant fragments in the misonidazole spectrum or the number of intensive fragments in the metronidazole spectrum (bottom, black) indicate this molecule is more fragile. Therefore, the radiosensitizers engage with very different bonding mechanism. The parent ion M^+^ at m/z 201 of misonidazole represents only a minor contribution to the mass spectrum and the main feature at m/z 45 can be assigned to the [CH_2_OCH_3_]^+^ fragment and corresponds to a part of the tail T_2_. The other noticeable feature is represented by the group at about m/z 155 assigned to the [M-NO_2_]^+^ fragment. All in all the [CH_2_OCH_3_]^+^ fragment and the group at about m/z 155 contribute to about 50% of the spectrum at this photon energy.

As for as the comparison with the 2-NI and 4(5)-NI spectra reported in Figure 1, it is noticeable that the loss of the NO group ([M-NO]^+^ with m/z 141 and 171 in the metronidazole and misonidazole, respectively) or the correlated product NO^+^ (m/z 30) appear to be, if any, a minor channel.

Based on the observations from the mass spectra in Figure 5, in the PEPICO and AE measurements we concentrated our attention on the parent ion, the fragments corresponding to [M-NO_2_]^+^ and [M-HONO]^+^ and the m/z 45 fragment which may correspond to the tail [HOCH_2_CH_2_]^+^ in metronidazole and a section of the tail [CH_2_OCH_3_]^+^ in the misonidazole. In the case of the metronidazole we also investigated two other fragments at m/z 81 and 54. A detailed report of the AE and PEPICO measurements relative to all the fragments observed in the mass spectra will be reported in a separate publication (Bolognesi, in preparation).

**Figure 6:**
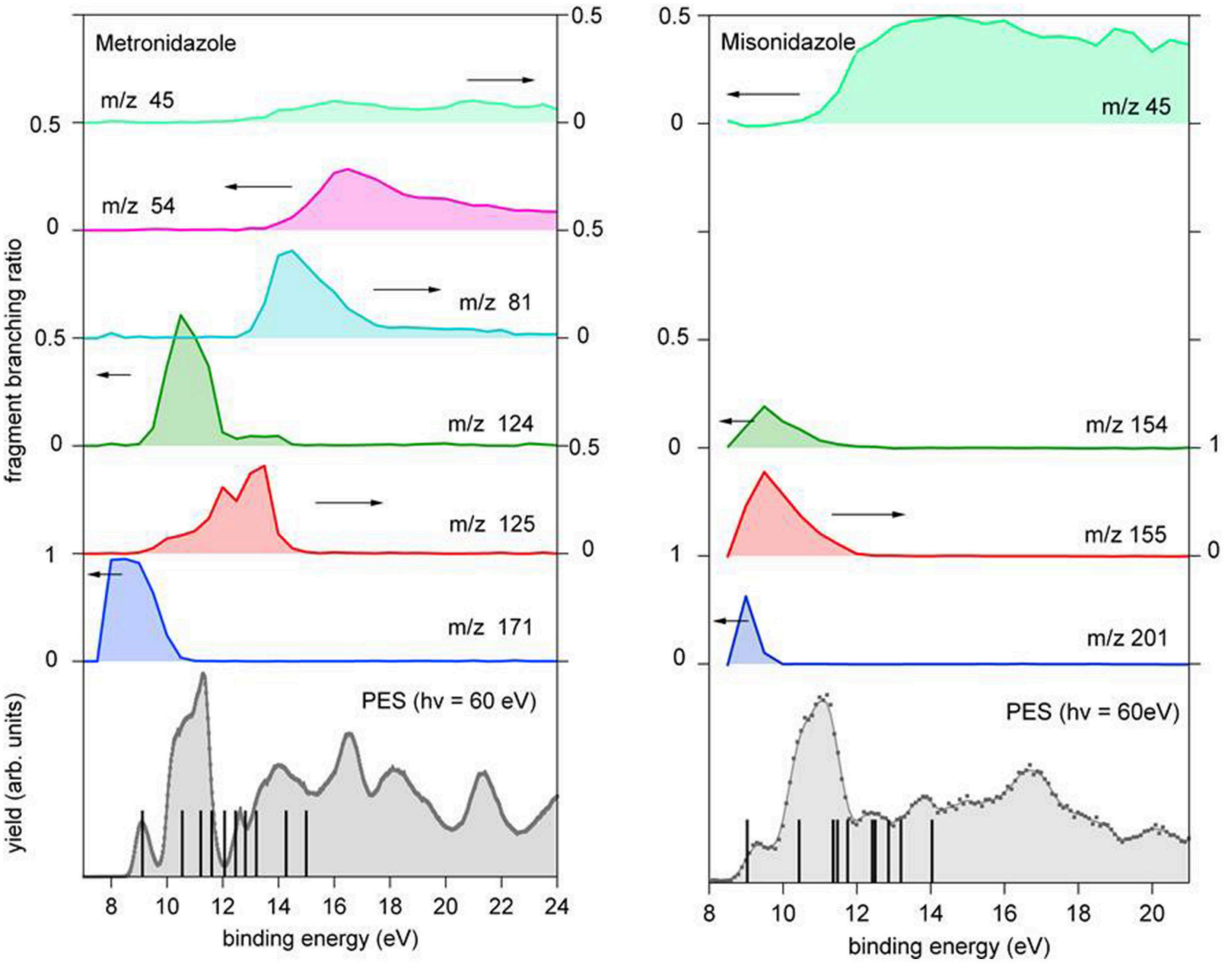
Branching ratio for the parent ion and a few fragments of metronidazole (left panel) and misonidazole (right panel) reported vs. the binding energy. The scale of the different branching ratios is indicated by the arrows. At the bottom of each figure the photoelectron spectrum of the molecule is reported. The vertical bars represent the binding energy of the cation states calculated using the outer valence Green function OVGF76-311++G** method (von Niessen et al., 1984), see Supplementary Material.

The experimental and calculated AE values are collected in Table 1, while the branching ratio of the parent ion and different fragments derived from the PEPICO measurements are shown in Figure 6. In the bottom panel of the same figures the photoelectron spectrum of each molecule measured at 60 eV is reported. The energies of the cation states calculated by the outer valence Green function OVGF6-311++G^**^ method (von Niessen et al., 1984) up to 15 eV are reported in Figure 6 and tabulated in Table 2SM. The spectroscopic pole strengths calculated in the Green's function model are in the range of 0.85–0.91, suggesting that the independent particle picture is a good approximation in this energy region. In the region of BEs higher than 15 eV electronic configurations with relaxation, two-hole-one-particle (2h-1p) and higher excitations may dominate the cationic states. These contributions, which represent the electronic correlation and relaxation, make the one-particle picture of the cationic states and/or vertical ionization process no more good approximations in this region. As a result, we concentrate on the outer valence region of the compounds in this section.

In both molecules the parent ions are possibly produced only via the ionization of the electrons on their highest occupied molecular orbital (HOMO) states. In the case of misonidazole the production of the parent ion competes already at threshold with dissociation channels involving the NO_2_ and HONO losses, see also Table 1. The same channels are observed in the metronidazole at about 1 eV above the ionization threshold with the HONO fragment loss having its maximum branching ratio in the energy region of HOMO-1 to HOMO-3 orbitals The NO_2_ loss channel is characterized by an AE very close to the one of the HONO loss, but becomes more effective at BE > 12 eV. The channel leading to the formation of the [CH_2_OCH_3_]^+^ fragment, which appears to be the dominant channel in the fragmentation of misonidazole, has a measured AE in the proximity of the BE of the HOMO-1 state and already at a BE of 12 eV its branching ratio is about 0.5. The channel leading to the m/z 45 fragment in metronidazole, which corresponds to the loss of the tail bound to N1 in the imidazole ring, displays a higher AE (about 12.09 eV), but a lower branching ratio (maximum value of about 0.1). The AE of m/z 124 and 125 fragments measured by Guo et al. (2012) are also reported in Table 1. The two sets of experimental data agree within their respective uncertainties.

## Discussion

The mass spectrum of the metronidazole molecule has been previously measured by 75 eV electron impact (Linstrom and Mallard, 2008) and at a few photon energies between 9.5 and 13 eV by Guo et al. (2012) in the m/z range 100–180. All the previously observed fragments of metronidazole in the electron impact spectrum are also present in Figure 5. However, we noted that slightly different intensities for the bands centered at approximately m/z 125 and 81, respectively, are observed in this spectrum. In the outer valence region, the measurements by Guo et al. (2012) are consistent with the present PEPICO experiments. At 9.5 eV only the parent ion is produced in the photoionization event, while at 11 eV fragments corresponding to the NO2 and HONO losses at m/z 124 and 125 are observed as well as at the highest photon energy used (13 eV) also the fragments corresponding to m/z = 126 and 127. Pandeti et al. (2017) observed the loss of C2H4O (44 Da) at position N1 followed by the NO2 loss as the dominant fragmentation channels in a collision induced dissociation experiment of protonated metronidazole. Such a process in the present case would lead to prominent features at m/z 127 and 81, respectively. While the feature at m/z 81 is clearly observed in the mass spectrum in Figure 5, the other one seems to give a minor contribution to our spectrum.

Less information is available for the mass spectrum of misonidazole. Recently Feketeova et al. (2014) presented fragmentation spectra of protonated misonidazole in the m/z range 60–205 obtained by collision and electron induced dissociation experiments. The assignment of the features observed in Figure 5 has been done according to that work and for the low m/z region not covered by the study of Feketeova et al. (2014) the results of the competitive fragmentation modeling method (Allen et al., 2014) have been employed to assist the analysis.

Very rare photoelectron spectrum (PES) studies are available for metronidazole and misonimidazole. The PES of metronidazole was measured and interpreted by a comparison with a series of spectra of simpler methylnitroimidazoles by Kajfez et al. (1979). The spectrum was measured with a HeI discharge lamp with a resolution of about 35 meV. Despite the lower resolution all the features assigned by Kajfez et al. (1979) are also visible in the present study as given in Figure 6 (see Table 3SM). The present measurement extends over a broader binding energy range up to 25 eV. To our knowledge no previous photoelectron spectrum of misonimidazole has been reported in the literature. The charge densities of the molecular orbitals in Table 4SM indicate that the HOMO is a π orbital located above and below the imidazole ring plane, while in the case of the HOMO-1 a contribution of σ type between C14 and C18 exists. In the comparison of the two experimental spectra the HOMO of the misonidazole appears to be stabilized (the BE being about 200 meV higher than in the metronidazole) while the theoretical predictions sets the BE of the HOMO of misonidazole about 100 meV below the metronidazole one, see Table 2SM. However, the differences are well within the accuracy of the approximation of the used theoretical method and the experimental uncertainties.

**Figure 7:**
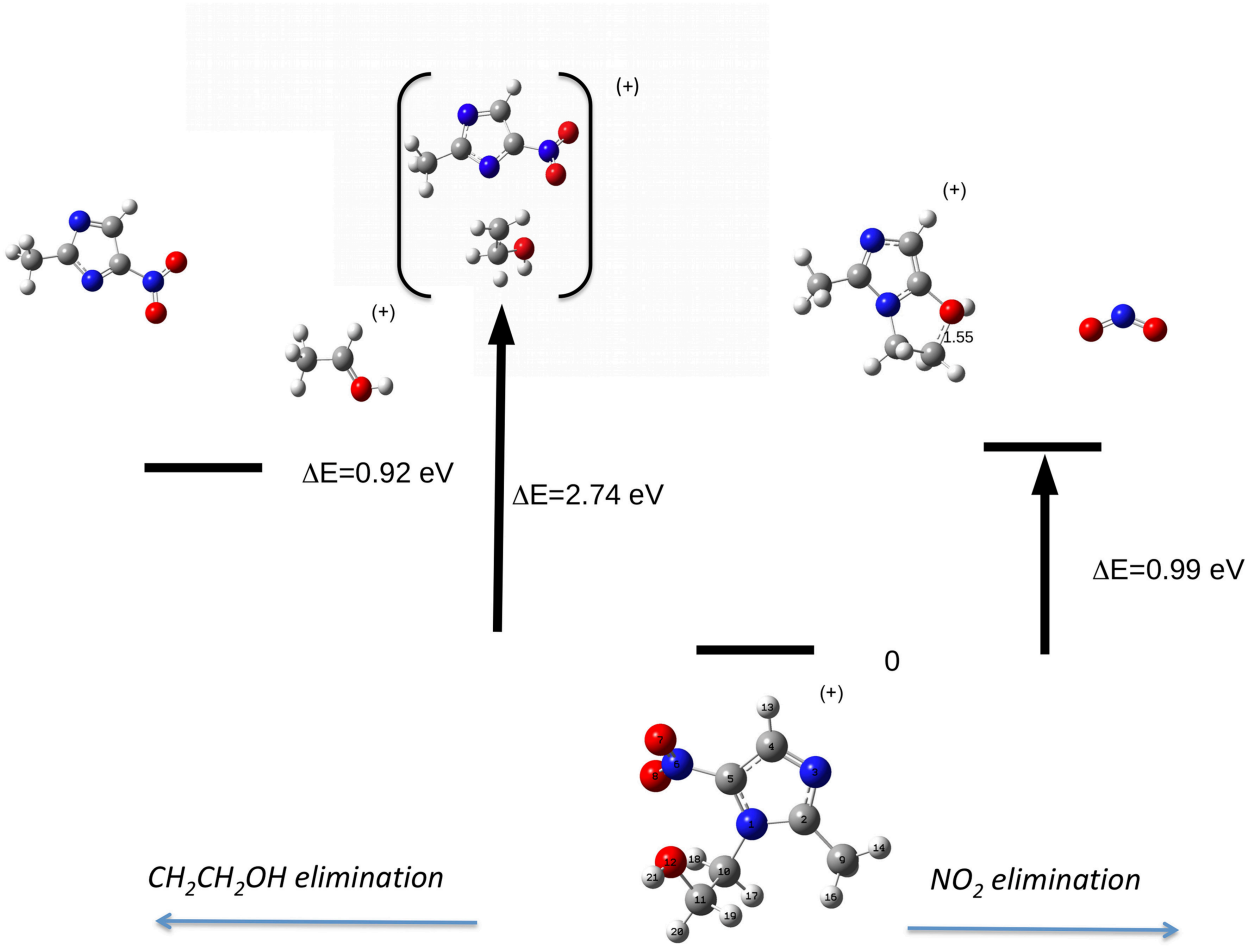
Fragmentation pathways in metronidazole leading to the NO_2_ (right panel) and tail CH_2_CH_2_OH (left panel) losses. The reaction coordinate in the right panel is R_C5−N6_, while in the left panel is R_N1−C10_. While in the first process the charge stays on the formed by-cyclic structure, in the second one the charge is on the smaller fragment. The energy values reported refer to the IP of metronidazole.

Let's now discuss the AEs and the ion yields determined in the energy selected PEPICO experiments. In the case of the metronidazole the parent ion (m/z 171) is observed (Figure 6) only in the region of the HOMO orbital. Already at about 1 eV above the ionization potential, IP, the state selected mass spectrum is dominated by the m/z 125 and 124 fragments, which correspond to the NO_2_ and HONO losses, respectively. The process leading to the NO_2_ elimination has been simulated. In this simulation as well as in all the others discussed later on in the text the full potential energy surface along the possible reaction coordinate has been explored. To simplify the representation in the figures only the transition states (TS) and the final optimized geometries of the products have been reported together with the relevant energies referred to the calculated adiabatic IP. The simulations summarized in Figure 7, right panel, shows that the process leading from the parent ion in its ground state to a charged fragment with m/z 125 and the NO_2_ elimination needs to overcome a barrier of about 1 eV. The calculated AE is in satisfactory agreement with the measured value. The simulation also indicates that the NO_2_ elimination leads to the formation of a by-cyclic structure in which O12 is bound to C5. This can be explained by considering as a starting configuration the conformer II (see Table 1SM) with the O12H group oriented toward the nitryl group.

This can be rationalized considering that the scans that lead to the identification of the conformer of minimum energy (see Figure 3), indicate that within an energy range of a few meV several conformers exist. As already mentioned, this represents a severe challenge for the simulations, because the use of a conformer as a starting point of a scan may strongly influence the reaction path. Guo et al. (2012) studied the NO_2_ loss and found that the cleavage of the C5-N6 bond is accompanied by an intramolecular hydrogen transfer. These authors considered three different formation pathways starting by two conformations of the parent ion, which differ by the orientation of the O12H hydroxil group with respect to the nitryl group. In all cases the transition states involve the formation of a by-cyclic structure with energy barriers between 1.1 and 1.3 eV, which is consistent with the one calculated in the present work.

The same authors (Guo et al., 2012) proposed similar reactions for the HONO loss. When the hydroxyl group is located at the same side of the nitryl one the H atom of the hydroxyl group is transferred to the nytril one and an intramolecular ring-closing reaction occurs via the formation of the C11O12-C5 bond. When the hydroxyl group is located at the other side one H atom migrates from C10 to the nytril group. The calculated energy barrier of 0.72 eV and AE = 9.20 eV for the first route are closer to the observed experimental value, thus it has been suggested as the most likely (Guo et al., 2012). Our extensive search for the transition state leading the HONO loss failed. The formation of the fragment at m/z 124 via the two successive losses of NO_2_ and H lead to a calculated AE of about 17 eV, i.e., more than 7 eV higher than the experimental observation. Thus, while our experimental observation is in agreement with the one by Guo et al. (2012) and consistent with the predicted value by the same authors we can't confirm theoretically the reaction pathway they proposed.

On the left part of Figure 7 the path leading to the loss of the tail T_1_ (CH_2_CH_2_OH, 45 Da) by the C10-N1 rupture is illustrated. The simulation shows that at the rupture of the bond the formed structure is CH_3_CHOH, i.e., a H migration occurred. In this process the charge might be localized either on the heavier fragment, which includes the imidazole ring, or on the tail. Considering the relative intensity of the peaks at m/z 126 and 45 in Figure 5, it seems that the process leading to the CH_3_CHOH ^+^ fragment is the most likely one. The appearance energy of this latter process has been calculated to be 11.34 eV, i.e., 2.74 eV above the IP. The predicted value is about 1 eV lower than the observed one. Guo et al. (2012) in the measurement at photon energy of 13 eV observed a tiny feature assigned to m/z 126 and calculated the AE of that fragment at 12.21 eV. Even though in our experiment the branching ratio for that fragment is vanishing, we calculated its AE and found a value of 12.16 eV consistent with the one by Guo et al. (2012).

It is interesting to note that in the scan of the potential energy surface no paths leading to the isomerization of the NO_2_ and the following NO loss, as observed in the 2- and 4(5)-NI, have been found. This is consistent with the experimental observation and represents a major difference in the fragmentation of the molecules studied here and their nitroimidazole model systems.

**Figure 8:**
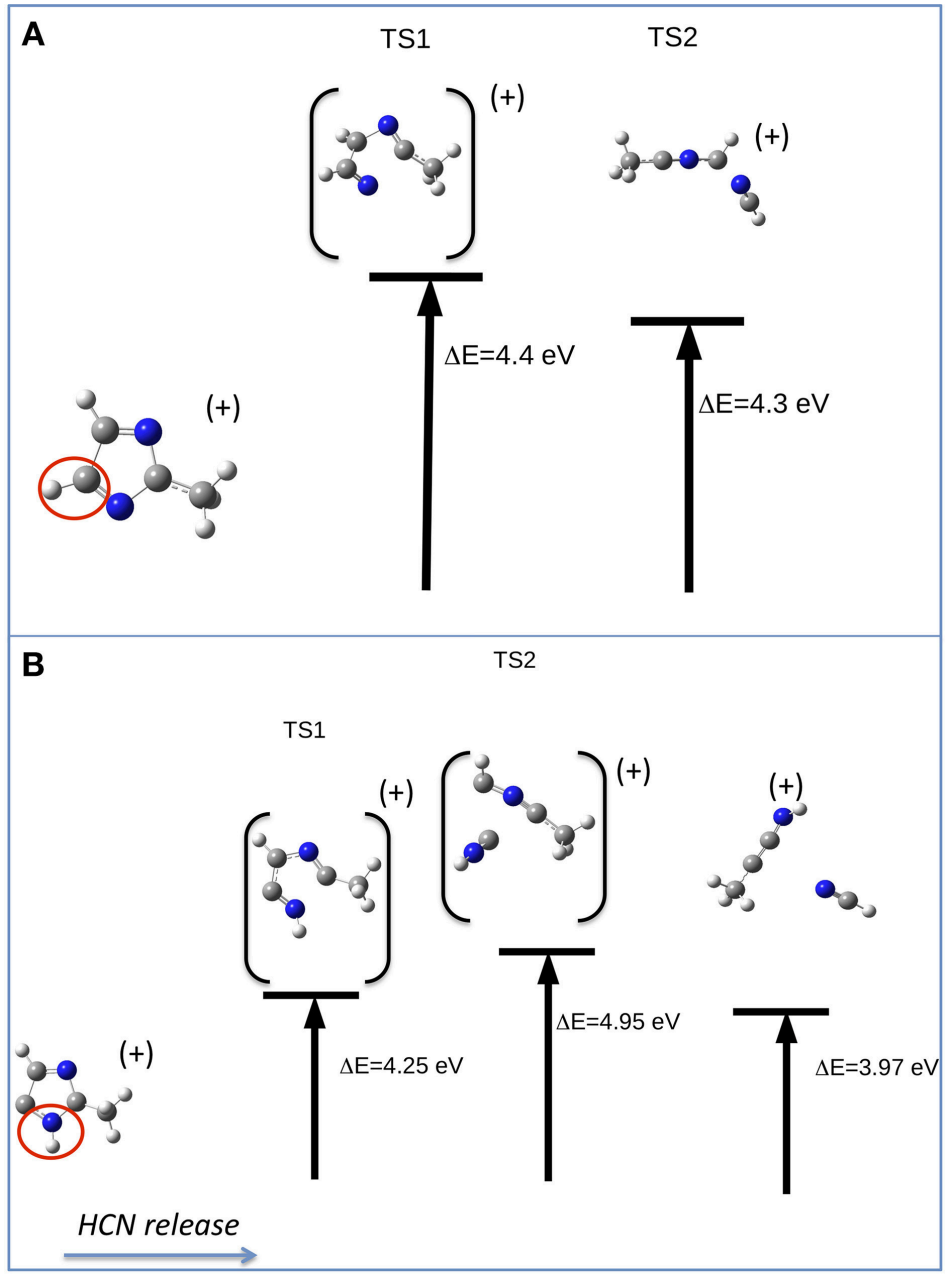
Fragmentation pathways in metronidazole leading from m/z 81 to the m/z 54 fragment and the neutral species HCN. The initial isomer is CH_3_CNCHCHN in pathway **(A)** and CH_3_CNHC_2_HN in pathway **(B)**. The ΔE values of the transition states refer to the IP.

The other relevant fragment in the mass spectrum of metronidazole is at m/z 81, which results from the successive losses of NO_2_ and CH_2_CHOH. Starting from the fragment m/z 125, the migration of H from the CH_2_OH group to the ring occurs, leading to a break of the double ring and subsequent elimination of CH_2_CHOH. The m/z 81 fragment, which can have two configurations with a H atom either bound to C5 or to N1 (Figures 8A,B, respectively), will further evolve with the formation of the fragment m/z 54 and the neutral species HCN (27 Da). Depending on the initial structure two interesting paths have been observed on the potential energy surface. In the first case (Figure 8A) a transition state at about 4.4 eV above the IP leads to the opening of the ring at the N3-C2 bond with the release of the HC4N3 group. In the second case (Figure 8B) one could have foreseen a direct elimination of HNC, but theoretical calculations demonstrate instead an unexpected mechanism. This involves two bond ruptures along the ring leading to HNC elimination as a first step. Then a closed structure with C4-C2 and C4-C5 bonds is formed (transition state at about 4.95 eV above the IP) and finally the HC5N1 group is lost. The predicted AEs for the m/z 54 fragment in both pathways are consistent with the experimental value, but the second one is closer to the experiment. Thus, it seems the most likely one.

**Figure 9:**
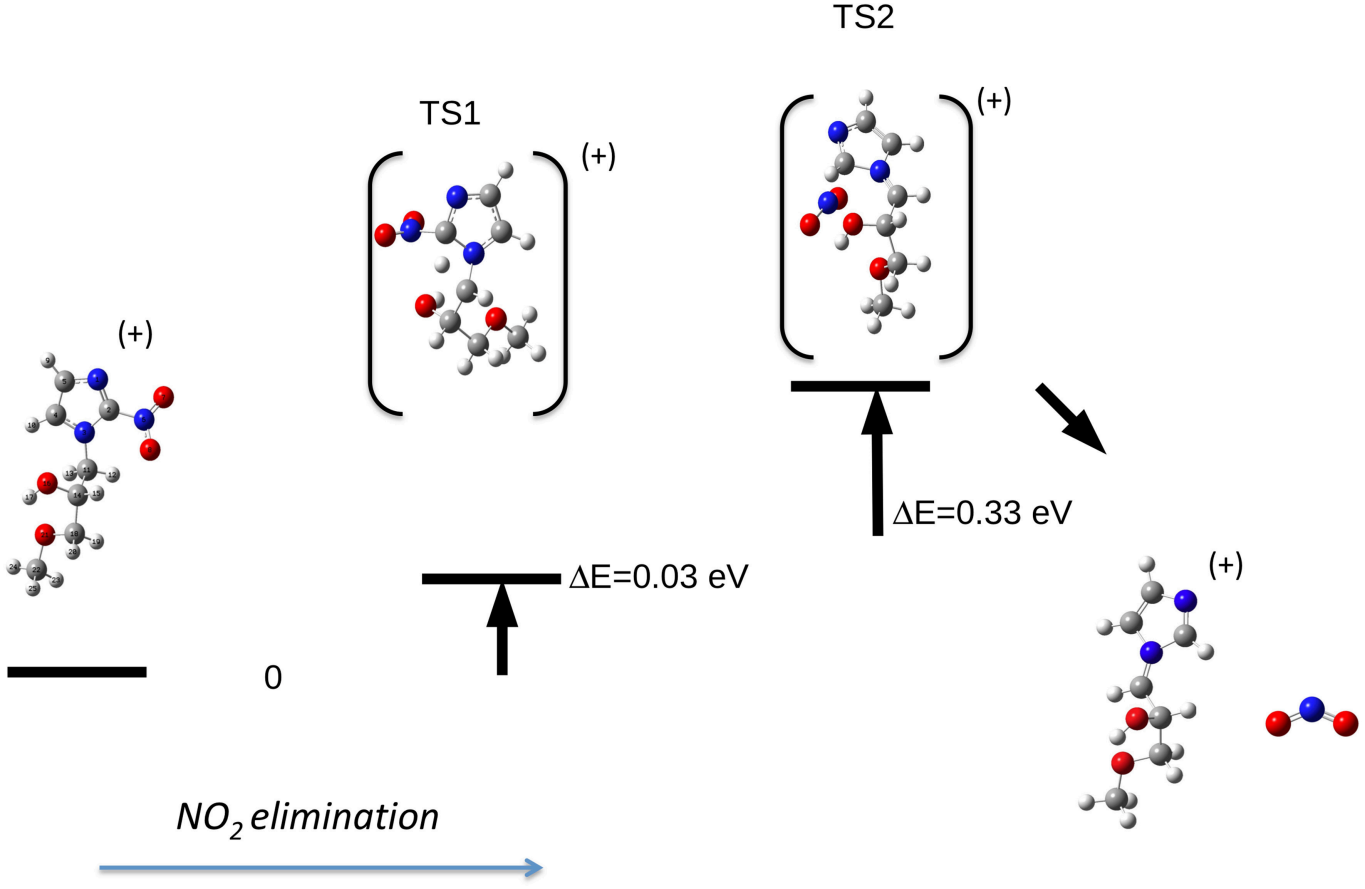
Fragmentation pathway in misonidazole leading to the NO_2_ loss and the m/z 155 fragment.

In the case of the misonidazole fragmentation the NO_2_ loss leading to fragment m/z 155 occurs via two transition states (see Figure 9) in which a H atom bond to C11 migrates from the tail to C2. The calculated AE is in satisfactory agreement with the measured value.

**Figure 10:**
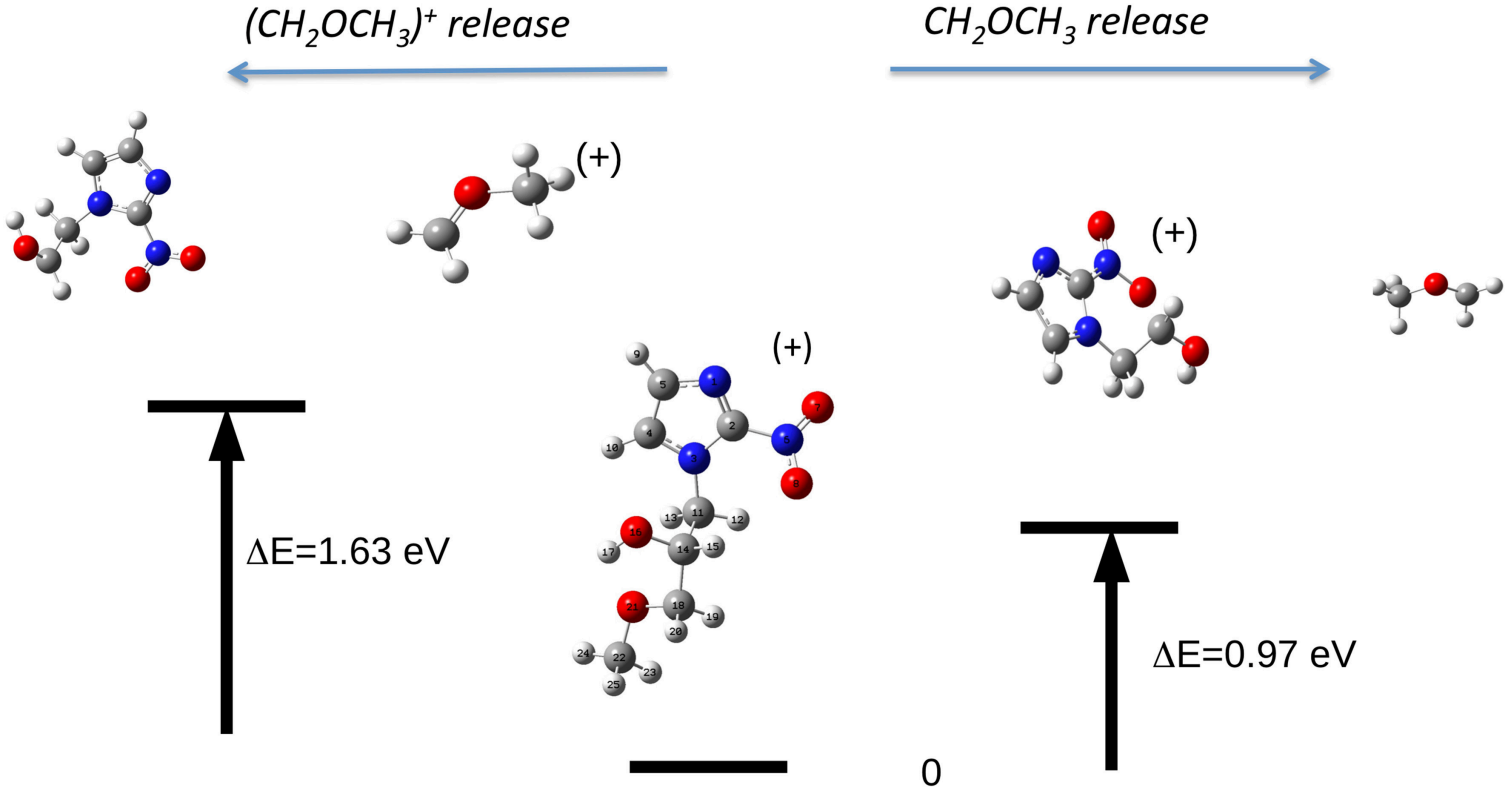
The fragmentation patterns in misonidazole leading to the loss of either neutral CH_2_OCH_3_ or [CH_2_OCH_3_]^+^ (m/z 45).

The reaction, that leads to the C_5_H_6_N_3_O_3_ (156 Da) and H_3_COCH_2_ (45 Da) moieties, can occur via two different paths of concerted mechanisms (Figure 10) depending on where the charge is localized. The path with the lower barrier (<1 eV) leaves the charge on the m/z 156 fragment, while the other path with a transition state of about 1.63 eV has the charge localized on the m/z 45 fragment. The mass spectrum at 60 eV (Figure 5) is dominated by this latter fragment and in the PEPICO spectra (Figure 6) the largest branching ratio is associated to this fragment, too. The observation that a channel not favored from an energetic point of view appears to be the dominant one in the experiment is unusual. It may be explained by the structure of the orbitals of the misonidazole. As seen in the bottom panel of Figure 6 and in Table 2SM no ionic states exist at about 1 eV above the IP and the charge of the π HOMO is mainly distributed above and below the ring (Table 4SM). Vice versa the HOMO-1 binding energy is calculated to be at about 1.4 eV above the IP and its charge distribution displays a contribution along the C14-C16 σ bond (Table 4SM). Thus, the removal of one electron from this orbital may weaken the bond and lead to the release of the charged tail ${\text{H}}_{3}{\text{COCH}}_{2}^{+}$.

It is interesting to observe that while in misonidazole the fragmentation channel leading to the [CH_2_OCH_3_]^+^ m/z 45, i.e., the loss of a charged part of the tail T_2_, appears to be the dominant channel with an AE close to the energy of the HOMO-1 orbital, the loss of the tail, [CH_2_CH_2_OH]^+^, in metronidazole has a definitely higher AE (>12 eV) and it is characterized by a small branching ratio in the PEPICO measurements. This indicates that this low-lying fragmentation channel in misonidazole represents the most efficient channel for energy dissipation.

## Conclusion

The photoinduced fragmentation of metronidazole and misonidazole molecules has been studied. The combination of photoionization mass spectrometry, photoelectron spectroscopy, photoelectron-photoion coincidence spectroscopy, and computational spectroscopy has been used to investigate and characterize the main reaction/fragmentation channels observed in the mass spectra, which correspond to the elimination of the NO2 and HONO group in both molecules and the formation of the [CH2OCH3]+ ion in misonidazole. The preferential elimination of the nitro-group (-NO2) in both molecules supports the hypothesis (Adams et al., 2012) that the radiosensitizer effect is due to the complex redox chemistry, which, occurring after the selective binding of the nitroaromatic compounds to hypoxic cells, involves the reduction of the nitro-group to an amine (-NH2).

The message that can be derived from the present results is that although both metronidazole and misonidazole contain imidazole ring in the backbone, the side branches of these molecules lead to different bonding mechanisms and properties. Metronitrodazole is very fragile and a complex fragmentation process follows the initial ionization. Misonidazole on the other hand is relatively robust. Ionization and fragmentation may occur simultaneously, as the intensity of the molecular ion in the mass spectrum is very small. Then the preferential loss of the [CH_2_OCH_3_]^+^ fragment, i.e., a part of the tail T_2_, acts as a protection of the ring against its opening and successive fragmentations. Therefore, if the therapeutic effect is linked to the nitroimidazole building block, then the efficient formation of the [CH_2_OCH_3_]^+^ fragment may help to protect the ring and to preserve the therapeutic effect of the compound.

More complex fragmentation can be linked to toxicity, thus from this point of view metronizadole, which displays a very rich mass spectrum, seems to have a higher toxicity than misonidazole.

The observation that the NO loss, the most relevant channel discussed in the previous studies (Bolognesi et al., 2016; Cartoni et al., 2018) in the 2-NI and 4(5)-NI molecules, is a minor, if any, channel in these compounds used in radiotherapy, prevents a direct extension of the chemical physics mechanisms identified in the building block molecules to the real drugs adopted in the clinical use. However the complex metabolism, that determines the biotransformation of NO and its related oxides *in vivo* (Kelm, 1999) does not allow to make definitive conclusions.

The results of our study, which has followed a bottom-up approach, indicate that translating the findings of chemical physics experiment to chemotherapeutic compounds is not an easy task. However considering that nitro compounds have found and are finding clinical application (Overgaard, 2011; Wang et al., 2018) the understanding of their chemical physics properties, of the specific mechanisms of the interaction between radiation and chemotherapy and of how the chemical radiosensitizers actually work at the molecular level is a challenge that cannot be neglected.

## Data Availability

The datasets generated for this study are available on request to the corresponding author.

## Author Contributions

PB, JC, RR, ST, BM, and LA performed the PEPICO experiments, while PB, MCC, and DC performed the AE measurements. ARC and AC performed the theoretical calculations and FW provided advices on the computational spectroscopy and interpretation of the spectra. PB, AC, JC, and MCC participated to the data analysis and interpretation. ARC, PB, and LA prepared the manuscripts. All the authors contributed to the interpretation of the results and the revision of the manuscript.

### Conflict of Interest Statement

The authors declare that the research was conducted in the absence of any commercial or financial relationships that could be construed as a potential conflict of interest.

## References

[B1] Adams G. E. Flockhart I. R. Smithern C. E. Stratford I. J. Wardman P. Watts M.E. (2012). Electron affinity sensitization:VII. A correlation between structure, one electron reduction potentials and efficiencies of nitroimidazoles as hypoxic cell radiosensitizers. Radiat. Res. 178, AV183-AV189. 10.1667/RRAV14.1 22870968

[B2] Allen F. Pon A. Wilson M. Greiner R. Wishart D. (2014). CFM-ID: a web server for annotation, spectrum prediction, and metabolite identification from tandem mass spectra. Nucleic Acids Res. 42, W94–W99. 10.1093/nar/gku436 24895432 PMC4086103

[B3] Berg J. M. Tymoczko J. L. Stryer L. (2012). Biochemistry, 7th Edn. New York, NY: W. H. Freeman and Company.

[B4] Blyth R. Delaunay R. Zitnik M. Krempasky J. Krempaska R. Slezak J. (1999). The high resolution gas phase photoemission beamline at Elettra. J. Electron. Spectrosc. Relat. Phenom. 101, 959–964. 10.1016/S0368-2048(98)00381-8

[B5] Bolognesi P. Casavola A. R. Cartoni A. Richter R. Markus P. Borocci S.. (2016). Communication: Position does matter: the photofragmentation of the nitroimidazole isomers. J. Chem. Phys. 145:191102. 10.1063/1.4967770 27875881

[B6] Boudaiffa B. Cloutier P. Hunting D. Huels M. A. Sanche L. (2000). Resonant formation of DNA strand breaks by low-energy (3 to 20 eV) electrons Science 287, 1658–1660. 10.1126/science.287.5458.1658 10698742

[B7] Cartoni A. Casavola A. R. Bolognesi P. Castrovilli M. C. Catone D. Chiarinelli J.. (2018). Insights into 2-and 4 (5)-Nitroimidazole decomposition into relevant ions and molecules induced by VUV ionization. J. Phys. Chem. A 122, 4031–4041. 10.1021/acs.jpca.8b01144 29652141

[B8] Castrovilli M. C. Bolognesi P. Cartoni A. Catone D. O'Keeffe P. Casavola A.. (2014). Photofragmentation of halogenated pyrimidine molecules in the VUV range. J. Am. Soc. Mass Spect. 25:351. 10.1007/s13361-013-0783-x 24385396

[B9] Chiarinelli J. Bolognesi P. Domaracka A. Rousseau P. Castrovilli M. C. Richter R. (2018). Insights in the dissociative ionization of glycine by PEPICO experiment. Phys. Chem. Chem. Phys. 20, 22841–22848. 10.1039/C8CP03473G 30151535

[B10] Derossi A. Lama F. Piacentini M. Prosperi T. Zema N. (1995). High flux and high resolution beamline for elliptically polarized radiation in the vacuum ultraviolet and soft x-ray regions. Rev. Sci. Instrum. 66, 1718–1720. 10.1063/1.1145828

[B11] Feketeova K. Albright A. L. Sørensen B. S. Horman M. R. Whit J. O'Hair R. A. J. (2014). Formation of radical anions of radiosensitizers and related model compounds via electrospray ionization. Int. J. Mass Spectr. 56, 365–366. 10.1016/j.ijms.2013.12.014

[B12] Frisch M. J. Trucks G. Schlegel H. B. Scuseria G. E. Robb M. A. Cheeseman J. R. (2009). Gaussian 09, Revision A.1. Wallingford: Gaussian, Inc.,

[B13] García Gómez-Tejedor G. Fuss M. C. (2012). “Radiation damage in biomolecular systems,” in Series: Biological and Biomedical Physics, Biomedical Engineering (Springer Netherland), 2012 10.1007/978-94-007-2564-5

[B14] Gonzalez C. Schlegel H. B. (1989). An improved algorithm for reaction path following. J. Chem. Phys. 90, 2154–2161. 10.1063/1.456010

[B15] Gonzalez C. Schlegel H. B. (1990). Reaction path following in mass-weighted internal coordinates. J. Phys. Chem. 94, 5523–5527. 10.1021/j100377a021

[B16] Guo H. Zhang L. Jia L. Qi F. (2012). Photon induced side-chain elimination of metronidazole: photoionization mass spectrometric and theoretical studies. J. Spectrosc. Dyn. 2:2.

[B17] Higgins G. S. O'Cathail S. M. Muschel R. J. McKenna W. G. (2015). Drug radiotherapy combinations: review of previous failures and reasons for future optimism. Cancer Treat. Rev. 41, 105–113. 10.1016/j.ctrv.2014.12.012 25579753

[B18] Kajfez F. Klansic Šunjic, V. L. (1979). Application of photoelectron spectroscopy to biologically active molecules and their constituent parts IV. methylnitroimidazoles. J. Heterocyclic Chem. 16, 529–531. 10.1002/jhet.5570160325

[B19] Kelm M. (1999). Nitric oxide metabolism and breakdown. Biochim. Biophys. Acta 1411, 273–289. 10.1016/S0005-2728(99)00020-1 10320663

[B20] Linstrom P. J. Mallard W. G. (2008). NIST Chemistry Webbook, Number 69. Gaithersburg, MD Available online at: http://webbook.nist.gov

[B21] Marr G. V. West J. B. (1976). Absolute photoionization cross-section tables for helium, neon argon, and krypton in the VUV spectral regions. At. Data Nucl. Data Tables 18, 497–508. 10.1016/0092-640X(76)90015-2

[B22] Michael B. D. O'Neill P. A. (2000). A sting in the tail of electron tracks Science 287, 1603–1604. 10.1126/science.287.5458.1603 10733428

[B23] Ortiz J. V. (1988). Electron binding energies of anionic alkali metal atoms from partial fourth order electron propagator theory calculations. J. Chem. Phys. 89,6348–6352. 10.1063/1.455401

[B24] Overgaard J. (2011). Hypoxic modification of radiotherapy in squamous cell carcinoma of the head and neck a systematic review and meta-analysis. Radiother. Oncol. 100, 22–32. 10.1016/j.radonc.2011.03.004 21511351

[B25] Pandeti S. Feketeova L. Reddy T. J. Abdoul-Carime H. Farizon B. Farizon M. (2017). Nitroimidazolic radiosensitizers investigated by electrospray ionization time-of-flight mass spectrometry and density functional theory. RSC Adv. 7, 45211–45221. 10.1039/C7RA08312B 30621427

[B26] Plekan O. Coreno M. Feyer V. Moise A. Richter R. De Simone M. (2008). Electronic state resolved PEPICO spectroscopy of pyrimidine. Phys. Scripta 78:058105 10.1088/0031-8949/78/05/058105

[B27] Rockwell S. Dobrucki I. T. Kim E. Y. Marrison S. T. Vu V. T. (2009). Hypoxia and radiation therapy: past history, ongoing research, and future promise. Curr. Mol. Med. 9, 442–458. 10.2174/156652409788167087 19519402 PMC2752413

[B28] Sonveaux P. Jordan B. F. Gallez B. Feron O. (2009). Nitric oxide delivery to cancer: Why and how? Eur. J. Cancer 45, 1352–1369. 10.1016/j.ejca.2008.12.018 19153039

[B29] von Niessen W. Schirmer J. Cederbaum L. S. (1984). Computational methods for the one-particle green's function. Comput. Phys. Rep. 1, 57–125. 10.1016/0167-7977(84)90002-9

[B30] Wang H. Mu X. He H. Zhan X. D. (2018). Cancer Radiosensitizers. Trends Pharmacol. Sci. 39, 24–28. 10.1016/j.tips.2017.11.003 29224916

[B31] Wardman P. Rothkamm K. Folkes L. K. Woodcock M. Johnston P. J. (2007). Radiosensitization by nitric oxide at low radiation doses. Radiat. Res. 167, 475–484. 10.1667/RR0827.1 17388699

[B32] Wiley W. McLaren I. H. (1955). Time-of-flight mass spectrometer with improved resolution. Rev. Sci. Instrum. 26, 1150–1157. 10.1063/1.1715212

[B33] Wilson W. R. Hay M. P. (2011). Targeting hypoxia in cancer therapy. Nat. Rev. Cancer 11, 393–410. 10.1038/nrc3064 21606941

[B34] Wong M. W. (1996). Vibrational frequency prediction using density functional theory. Chem. Phys. Lett. 256, 391–399. 10.1016/0009-2614(96)00483-6

[B35] Yoshikawa S. Caughey W. S. (1990). Infrared evidence of cyanide binding to iron and copper sites in bovine heart cytochrome c oxidase. Implications regarding oxygen reduction. J. Biol. Chem. 265, 7945–7958. 2159465

