# Supplementary material for: Radiation Damage Mechanisms of Chemotherapeutically Active Nitroimidazole Derived Compounds
Source: Front Chem. 2019 May 14;7:329. doi: 10.3389/fchem.2019.00329 (PMC6528692; doi:10.3389/fchem.2019.00329)
Supplement: Supplementary file 1 [file Data_Sheet_1.pdf]

Supplementary Material

Radiation damage mechanisms of chemotherapeutically active nitro compounds

J. Chiarinelli[1,2], A. Casavola[1], M.C. Castrovilli[1], P.Bolognesi[1], A. Cartoni[3,1], Feng Wang[4], R. Richter[5], D. Catone[6], S. Tosic[7] , B. P. Marinkovic[7], L. Avaldi[1]*

[1]CNR-Istituto di Struttura della Materia, Area della Ricerca di Roma 1, CP10 Monterotondo Scalo, Italy
[2]Dipartimento di Scienze, Università di Roma Tre, Via della Vasca Navale, Roma, Italy
[3]Dipartimento di Chimica, Sapienza Università di Roma, Roma, Italy
[4]Molecular Modelling Discovery Laboratory, Department of Chemistry and Biotechnology, Faculty of Science, Engineering and Technology, Swinburne University of Technology, Melbourne, Vic 3122, Australia
[5]Elettra-Sincrotrone Trieste, Strada Statale 14-km 163.5, Basovizza, Trieste, Italy.
[6]CNR-Istituto di Struttura della Materia, Area della Ricerca di Tor Vergata, Via del Fosso del Cavaliere, Roma,Italy
[7]Institute of Physics, Laboratory for Atomic Collision Processes, University of Belgrade, 11080 Belgrade, Serbia

1. Metronidazole : the minimum structures for the C10-C11 and N6-C5 rotations

Table 1SM: Parameters of the minimum structures for the C10-C11 and N6-C5 rotations of the metronidazole molecule. R_1 is the perimeter of the imidazole ring, R_2 is the perimeter of the "ring" (H16-C8-N3-C2-N6-O7) formed by the hydrogen bonding. R_A, R_B and R_C are the rotational constants relative to the three principal axis moments of inertia of the molecule.

Parameters	C10-C11 rotation Conformer I	N6-C5 rotation Conformer II
	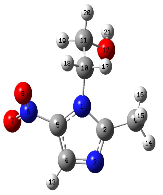	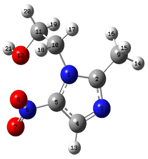
R_1	6.854	6.854
R_2	8.937	8.98
Bond length (Å) NO2	1.217	1.216
Bond angles (˚)	128.035	128.41
Dihedral angles (˚)	-36.078	-42.568
$<R^2>$ (a.u.)	1912.3820	1831.5461
μ (D)	4.0275	5.9189
ΔE (a.u.)	0.172325	0.172361
ZPE ($kcal.mol^{-1}$)	100.71124	100.64223
E + ZPE (a.u.)	-623.577188	-623.577331
E (Eh)	-623.7376868	-623.737719
ΔE(KJ/mol)	0.085	0.0
HOMO – LUMO gap (eV)	4.53956	4.834
Rotational Constants(GHz)		
R_A	1.27245	1.30075
R_B	0.84878	0.90767
R_C	0.60437	0.64224

2. The binding energies of the outer valence states of Misonidazole and Metronidazole

Table 2SM: Outer valence ionization energies (eV) of misonidazole and metronidazole calculated using the outer valence Green function OVGF76-311++G** method

Misonidazole			Metronidazole	
Orbital	OVG	Pole strength	OVG	Pole strength
HOMO	9.026	0.906	9.116	0.906
HOMO-1	10.434	0.901	10.541	0.903
HOMO-2	11.363	0.926	11.598	0.856
HOMO-3	11.475	0.856	11.205	0.911
HOMO-4	11.353	0.913	12.064	0.919
HOMO-5	11.747	0.901	12.457	0.907
HOMO-6	12.414	0.916	12.808	0.917
HOMO-7	12.495	0.908	13.201	0.924
HOMO-8	12.855	0.92	14.275	0.917
HOMO-9	13.189	0.925	14.268	0.893
HOMO-10	14.033	0.916	14.992	0.920

Table 3SM: Energy of the experimental features observed in the photoelectron spectra of metronidazole and misonidazole measured at 60 eV photon energy.

Metronidazole		Misonidazole
Present work	Kajfěz et al (1979)	Present work
9.08	9.10	9.23
10.47	10.34	10.63
	10.64	
11.31	11.34	11.04
12.63	12.63	12.33
13.53	13.80	13.74
14.01	14.23	
14.73	14.83	15.14
16.53	16.73	16.64
18.15		18.24
21.36		20.24
24.24		21.84

3. Representation of the Hatree-Fock molecular orbitals.

Table 4SM: representation of the charge density of the HF molecular orbitals of metronidazole and misonidazole

**Metronidazolo**	
HOMO	HOMO-1
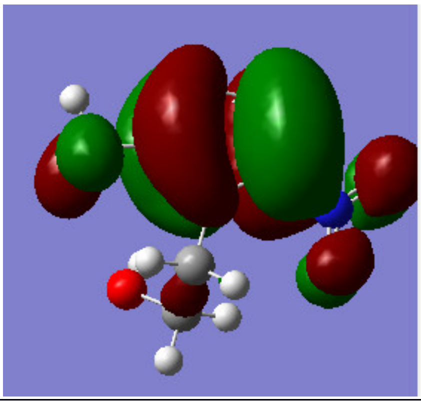	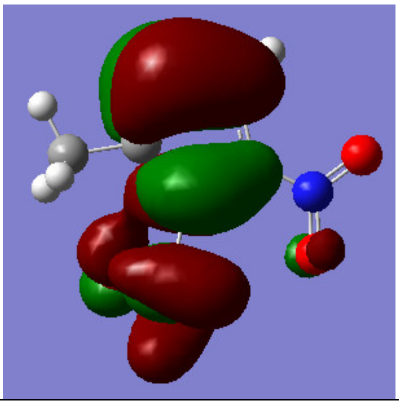
HOMO-2	HOMO-3
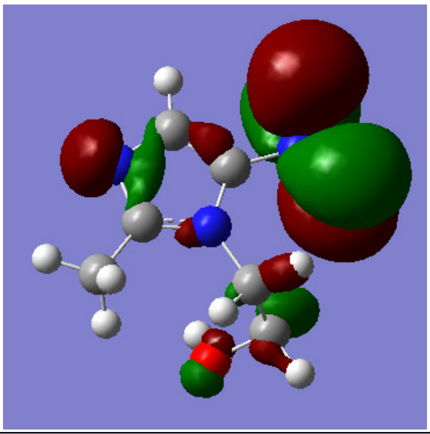	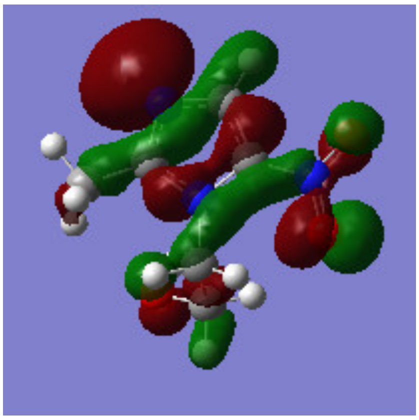

**Misonidazolo**	
HOMO	HOMO-1
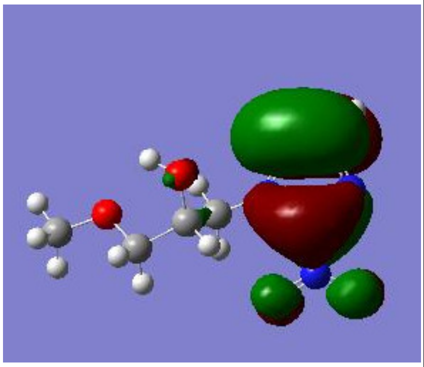	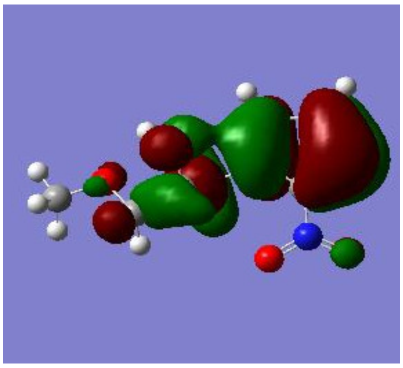
HOMO-2	HOMO-3
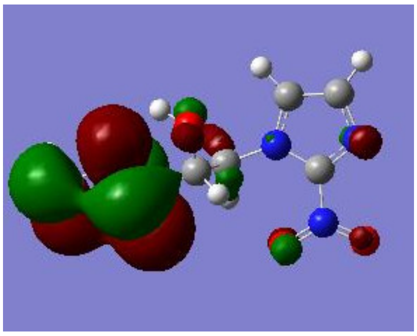	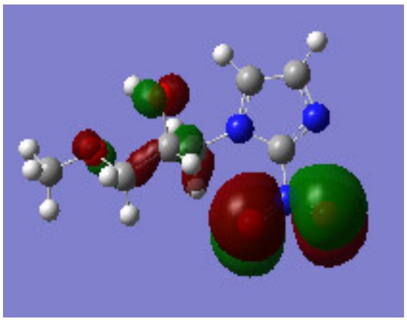

References

Kajfĕz, F., Klansic and L., Šunjic, V. (1979). Application of Photoelectron Spectroscopy to Biologically Active Molecules and Their Constituent Parts IV.Methylnitroimidazoles. *J. Heterocyclic Chem.*16,529-531
